# Current State and Challenges of the Global Outcomes of Dental Caries Research in the Meta-Omics Era

**DOI:** 10.3389/fcimb.2022.887907

**Published:** 2022-06-17

**Authors:** Dina G. Moussa, Paras Ahmad, Tamer A. Mansour, Walter L. Siqueira

**Affiliations:** ^1^College of Dentistry, University of Saskatchewan, Saskatoon, SK, Canada; ^2^Department of Population Health and Reproduction, School of Veterinary Medicine, University of California, Davis, CA, United States; ^3^Department of Clinical Pathology, School of Medicine, Mansoura University, Mansoura, Egypt

**Keywords:** dental caries, metagenomics, metatranscriptomics, metaproteomics, metabolomics, host-microbiome interactions, integrative multi-omics, bibliographic

## Abstract

Despite significant healthcare advances in the 21^st^ century, the exact etiology of dental caries remains unsolved. The past two decades have witnessed a tremendous growth in our understanding of dental caries amid the advent of revolutionary omics technologies. Accordingly, a consensus has been reached that dental caries is a community-scale metabolic disorder, and its etiology is beyond a single causative organism. This conclusion was based on a variety of microbiome studies following the flow of information along the central dogma of biology from genomic data to the end products of metabolism. These studies were facilitated by the unprecedented growth of the next- generation sequencing tools and omics techniques, such as metagenomics and metatranscriptomics, to estimate the community composition of oral microbiome and its functional potential. Furthermore, the rapidly evolving proteomics and metabolomics platforms, including nuclear magnetic resonance spectroscopy and/or mass spectrometry coupled with chromatography, have enabled precise quantification of the translational outcomes. Although the majority supports ‘conserved functional changes’ as indicators of dysbiosis, it remains unclear how caries dynamics impact the microbiota functions and vice versa, over the course of disease onset and progression. What compounds the situation is the host-microbiota crosstalk. Genome-wide association studies have been undertaken to elucidate the interaction of host genetic variation with the microbiome. However, these studies are challenged by the complex interaction of host genetics and environmental factors. All these complementary approaches need to be orchestrated to capture the key players in this multifactorial disease. Herein, we critically review the milestones in caries research focusing on the state-of-art singular and integrative omics studies, supplemented with a bibliographic network analysis to address the oral microbiome, the host factors, and their interactions. Additionally, we highlight gaps in the dental literature and shed light on critical future research questions and study designs that could unravel the complexities of dental caries, the most globally widespread disease.

## Introduction

**Figure 1:**
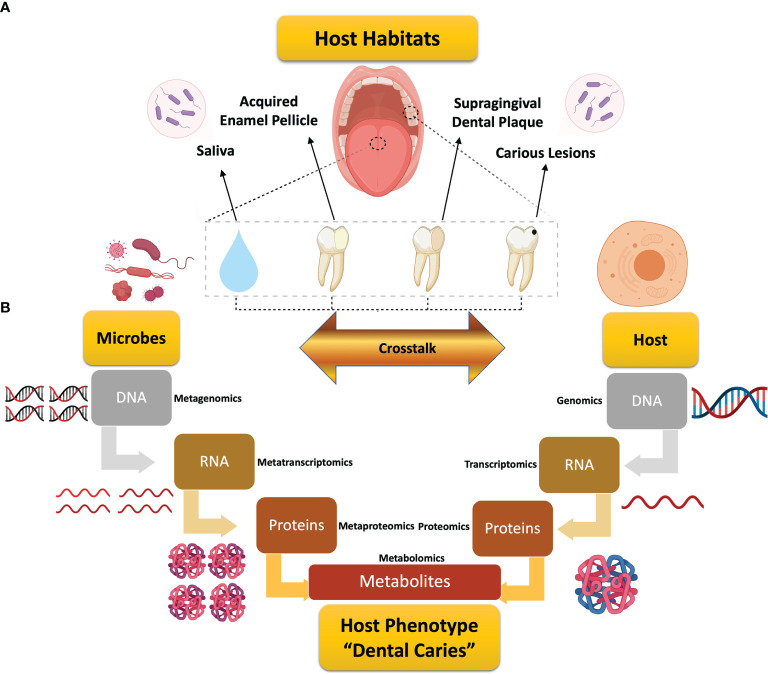
Schematic diagram depicting the review design and workflow for global assessment of omics studies in dental caries research. **(A)** Illustration of the scope of this review for the studied host habitats in dental caries research. **(B)** Illustrative representation of the microbiome- and host-related flow of information along the central dogma of biology from the genomic data to the end products of metabolism that could individually or jointly underlie the expression of dental caries. Each stage is associated with the corresponding systems biology tool, from genomics to metabolomics, with added “meta” prefix that implies “many” for the multispecies microbial communities. Metabolomics, the analytical tool for metabolites, embraces the microbial metabolism and microbial–host co-metabolism.

Dental caries remains the most common chronic disease globally, with corresponding exorbitant costs in healthcare budgets (GBD 2017 Disease and Injury Incidence and Prevalence Collaborators, 2018; United Nations General Assembly, 2011; World Health Organization, 2020). In 2018, the annual cost of dental care was nearly $136 billion in the United States alone, which represents 3.7% of the country’s total health care expenditures (U.S. Department of Health and Human Services, 2021). Dental caries presents as a complex, dynamic pH-driven disease, in which onset and progression are controlled by intricate host and microbial factors (**Figure 1**). Neither these factors, nor their crosstalk, have yet to be well-defined (Wright, 2010; Lamont et al., 2018; Rosier et al., 2018).

Over the years, etiological concepts of dental caries have been evolving to explain the onset and pathogenesis of this long-standing multifactorial disease. Despite different theories, caries is basically the outcome of dysbiotic changes in the oral biofilm community mediated by the host’s frequent intake of fermentable sugars and carbohydrates (Nyvad et al., 2013; Simon-Soro and Mira, 2015). As a result, a prolonged low pH environment continues to select for acid-tolerant microorganisms that favor the persistence of this disturbed ecosystem and, eventually, the initiation of demineralized carious lesions (Bradshaw and Lynch, 2013; Moynihan and Kelly, 2014; Sheiham and James, 2015; Boisen et al., 2021). Other host and behavioral factors, such as genetic factors, enamel defects, salivary flow and composition, oral hygiene, or dietary habits also contribute to caries development (Takahashi and Nyvad, 2011; Pitts et al., 2017; Bowen et al., 2018). Herein, we present the first all-inclusive critical review addressing both host and microbe, separated and integrated, contributions in dental caries research and comprehensively mapping the conducted studies along the omics hierarchy to provide a deep insight into the current understanding of the disease and the future research directions (**Figure 1**).

## Evolved Theories and Hypotheses for Dental Caries

**Figure 2:**
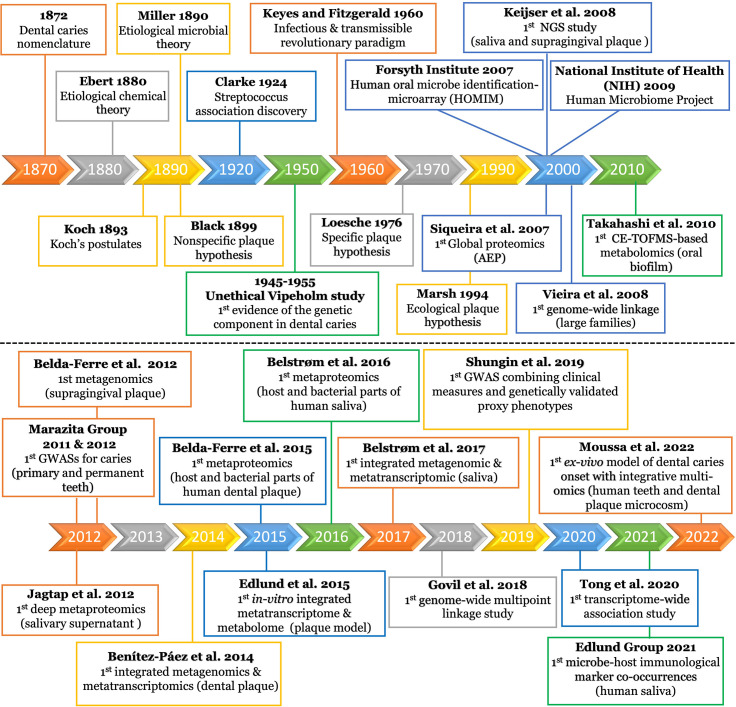
Timeline of milestones in dental caries research since emergence. The top panel displays the research milestones from the beginning of the field until 2010. The timeline displays selected intervals guided by the sporadic distribution of the earlier research events. The bottom panel displays the research events from 2010 to the present, using a one year interval. The details of each research event is displayed in the corresponding box of the timeline interval. AEP, acquired enamel pellicle; NGS, next generation sequencing; CE-TOFMS, capillary electrophoresis-time of flight mass spectrometer; GWAS, genome-wide association study.

The dental caries research era began in the latter part of the nineteenth century (**Figure 2**). Earlier, dental caries was described as a pathological condition of teeth, in which a portion of their structures became impaired, disintegrated, and decomposed. The disease was also described as “mortification”, “gangrene”, and/or “nigritis ossium” until the terminology “dental caries” gained acceptance and became commonplace for most scientists (Koch, 1872).

### The Chemical Theory

It was not clear if this pathological condition is wholly chemical or vital process (Chase, 1873). The American Dental Society attributed dental caries chiefly to food remaining on and between the teeth. They justified their claim basing it on fewer instances of dental caries in the lower front teeth that are tightly closed to food entrance (Stewart, 1878). Still, the debate remained as to whether the causative factors were local or general or, eccentrically considered as a malignant disease (Black, 1880) until the Chemical Theory was proposed. This theory attributed the destruction of tooth substance to acids originating from special bacterial species or secretions of the mucous membrane and/or of the parotid glands (**Figure 2**) (Ebert, 1880).

### The Nonspecific and Specific Plaque Hypotheses

The microbial etiology of dental caries was clearly postulated by Miller in 1890 (Hopkins, 1899) and, for several decades, it was commonly accepted that caries was caused by the nonspecific overgrowth of bacteria in dental plaque. The Nonspecific Plaque Hypothesis (NSPH) postulated that only the quantity of plaque determined the level of pathogenicity (**Figure 2**) (Black, 1899). In 1924, the European microbiologists were the first to discover association between *Streptococcus* and carious lesions, and J. Clarke named it *Streptococcus mutans (S. mutans)* (Clarke, 1924). Keyes and Fitzgerald changed the NSPH paradigm with the “Keyes and Fitzgerald Revolution”, which described dental caries as an infectious and transmissible disease conducted by *S. mutans* (**Figure 2**) (Fitzgerald and Keyes, 1960). This bacterial strain gained widespread attention within the scientific community and was considered the main caries pathogen from which, the Specific Plaque Hypothesis (SPH) was stemmed (**Figure 2**) (Loesche, 1976; Loesche and Grenier, 1976).

### The Ecological Plaque Hypothesis

The advancement of molecular methods enabled scientists to observe that carious lesions could occur in the absence of *S. mutans*. Consequently, the Ecological Plaque Hypothesis (EPH) emerged to emphasize a mixed bacterial-ecological etiology of dental caries (**Figure 2**) (Marsh, 1994). EPH combined the key concepts of the earlier two hypotheses (**Figure 2**) and proposed that dental caries is the result of an imbalance in the microflora dominated by acid-tolerating bacteria due to ecological selection (Kleinberg, 2002; Marsh, 2003). Given the pronounced variability in microbiota across individuals, different microbial combinations were found to have essentially the same functional profile. Therefore, focusing on ‘what bacteria are doing’ was revealed to be important in controlling the disease, regardless of the specific microbial compositions involved in the process (Takahashi and Nyvad, 2011; Simon-Soro and Mira, 2015).

### Genetic Factors

EPH was criticized for not considering the host genetic component in the caries process (Rosier et al., 2014). Genetic contributions were initially observed in the Vipeholm study; although considered unethical, as intellectually disabled patients were fed large amounts of cariogenic snacks over several years to provoke caries, genetic resistance was a striking observation with 20% of the subjects not developing any carious lesions (Gustafsson et al., 1954). This genetic resistance was further supported by observations of lower caries prevalence in parents and siblings of the subjects showing genetic resistance, when compared to the rest of the population (Böök JAaG, 1953). Since then, additional studies have provided evidence that there is a genetic contribution to dental caries, with certain population groups being more genetically susceptible to dental caries (**Figure 2**) (Bretz et al., 2005; Wang et al., 2013; Opal et al., 2015).

## Molecular Microbial Analyses in Caries Research

### Pre-Omics Era

Traditional culturing and biochemical characterization have provided considerable knowledge regarding caries microbial associations. However, these approaches are limited since >50% of the oral microbiota is uncultivatable (Paster and Dewhirst, 2000; Paster et al., 2001; Dewhirst et al., 2010). Accordingly, molecular techniques were developed to define the microbes based on their genomic identification, thus circumventing this culturing hurdle (Leser et al., 2002). The vast majority of these molecular techniques are based on the 16S ribosomal RNA gene (16S rRNA), which can be analyzed after DNA extraction from bacteria. 16S rRNA consists of highly conserved regions, allowing universal primers to target all bacteria, interspersed with species-specific hypervariable regions (Patel, 2001). A polymerase chain reaction (PCR) with universal primers matching the conserved regions can amplify 16S rRNA amplicons from wide range of bacterial species. Denaturing gradient gel electrophoresis exploits the melting behaviors of different 16S rRNA sequences, reflected distinctly through a polyacrylamide gel, to discriminate between these species. Although the 16S rRNA gene has been the most widely used phylogenetic marker for studying oral microbial communities for almost two decades (Ahn et al., 2011), limitations have been noted. For instance, PCR-based techniques, including multiplex and real-time quantitative PCR, were only capable of probing up to 10 species out of the hundreds of species comprising the oral microbial community (Terefework et al., 2008). Moreover, gel electrophoresis is not a quantitative technique and can merely differentiate a few taxa in a given sample (Muyzer et al., 1993).

### Early Omics Era

The first tool capable of high-throughput characterization of oral microbiota were 16S rRNA gene microarrays (Wagner et al., 2007). These microarrays utilize species-specific 16S rRNA gene sequences as probes to target complementary sequences. The Forsyth Institute pioneered the development of the Human Oral Microbe Identification microarray (Ahn et al., 2011) that is capable of detecting 300 oral species in a single hybridization. Microarrays have revealed a high degree of previously uncharacterized diversity in microbial communities, with healthy subjects having more diverse microbial communities when compared to caries-active subjects (Preza et al., 2009). Despite the efficiency and reproducibility of microarrays, they have the probe-wise limitation, in that only microorganisms targeted by the probes can be detected (Liu et al., 2010; Nyvad et al., 2013).

## Meta-Omics Studies: Search Strategy and Network Mapping

A continually increasing number of omic studies are utilizing high-throughput microarrays and sequencers as well as mass spectrometric technologies. Therefore, we performed a bibliographic analysis to create a network map of the frequency and co-occurrence of keywords associated with omic-related publications in dental caries research. This approach enables articulating a vast number of publications and provides a simplistic graphical representation for extracting insights from data (Zimmer, 2006; van Eck and Waltman, 2010; Ahmad et al., 2020; Zyoud and Al-Jabi, 2020). The bibliographic method adopted in this work has a descriptive, exploratory nature that depends on a quantitative and qualitative investigatory approach (mixed method) (Pereira et al., 2019). This bibliographic networking enabled us to analytically assess the global output of dental caries research to identify trends, gaps, and alignment of methodological approaches. Our focus was on modern omics research studies of microbes, host factors, and their interactions in dental caries research.

### Search Strategy and Data Extraction

On November 1^st^, 2021, the dental caries literature was screened *via* Elsevier’s Scopus as it provides the largest abstract and citation database of peer-reviewed scientific literature (Falagas et al., 2008; Kulkarni et al., 2009; Mongeon and Paul-Hus, 2015; Ahmad et al., 2019). The first step was to define search words that would be used to screen the literature; search words included dental caries terms (n=12) and omics-related terms (n=44). A search of article titles, abstracts and/or keywords for combinations of these terms was completed. The number of publications matching each combination of dental caries- and omic-related terms are listed in (**Table 1**).

**Table 1:** The search terms and their combinations between dental caries- and omics-related terms used for data extraction from Elsevier's Scopus database.

Combined with		Caries-related Search Terms (n = 12)								
Omics-related Search Terms (n = 44)		Caries	Carious	Cariogenic	Tooth* decay	Tooth* cavity	Dental decay	Dental cavity*	Cariology	Cariogenesis
1	Next generation sequencing	71	10	13	2	0	1	0	0	1
2	Expression array	0	0	0	0	0	0	0	0	0
3	High throughput sequencing	86	10	9	4	0	0	0	0	0
4	Pyrosequencing	52	5	4	1	0	1	1	0	0
5	Amplicon	68	10	9	2	0	2	1	0	1
6	Genotyping array	0	0	0	0	0	0	0	0	0
7	Microarray	97	12	17	4	0	0	0	0	2
8	metagenomic*	82	9	13	3	0	0	0	0	2
9	metagenome	67	5	7	3	0	1	1	0	1
10	Shotgun sequencing	9	1	0	0	0	0	0	0	6
11	16s rRNA	253	42	29	12	0	4	3	2	0
12	16s RNA	9	1	0	0	0	0	0	0	0
13	Genomic*	306	20	38	14	0	2	1	2	8
14	Genome	292	16	55	19	0	5	4	0	10
15	Genome-wide	62	4	2	7	0	0	0	0	5
16	Microbiome	425	36	70	26	0	8	3	0	4
17	Metatranscriptomic*	16	4	1	4	0	0	0	0	0
18	Metatranscriptome	10	1	2	1	0	0	0	0	0
19	Transcriptomic*	38	5	12	4	0	0	0	0	1
20	Transcriptome	64	8	20	7	0	1	0	0	1
21	Metaproteomic*	10	1	0	1	0	0	0	0	0
22	Metaproteome	5	0	0	1	0	0	0	0	0
23	Proteomic*	103	13	21	9	0	1	0	0	2
24	Proteome	68	9	12	3	0	1	0	0	2
25	Metabolomic*	55	3	5	1	0	1	0	0	1
26	Metabolome	26	1	3	0	0	0	0	0	0
27	Nuclear Magnetic Resonance	333	27	43	13	2	2	13	0	0
28	Mass spectrometry	291	0	98	13	0	2	9	0	1
29	Meta-omics	2	0	0	0	0	0	0	0	0
30	Omic*	23	1	2	3	0	1	0	0	0
31	GWAS	22	1	0	3	0	0	0	0	3
32	RNAseq	2	0	0	0	0	0	0	0	0
33	RNA-seq	19	4	7	2	0	0	0	0	0
34	DNAseq	0	0	0	0	0	0	0	0	0
35	DNA-seq	0	0	0	0	0	0	0	0	0
36	Degradomic	1	0	0	0	0	0	0	0	0

The search strategy covered the range of “article title, abstract, authors keywords”. A total of 12 caries-related search terms were implemented versus 44 omics-related search terms. The entries with ‘*’ indicates that the search term was also employed in its plural form, for a total of 44 omics-related search terms.

### Network Analysis

**Figure 3:**
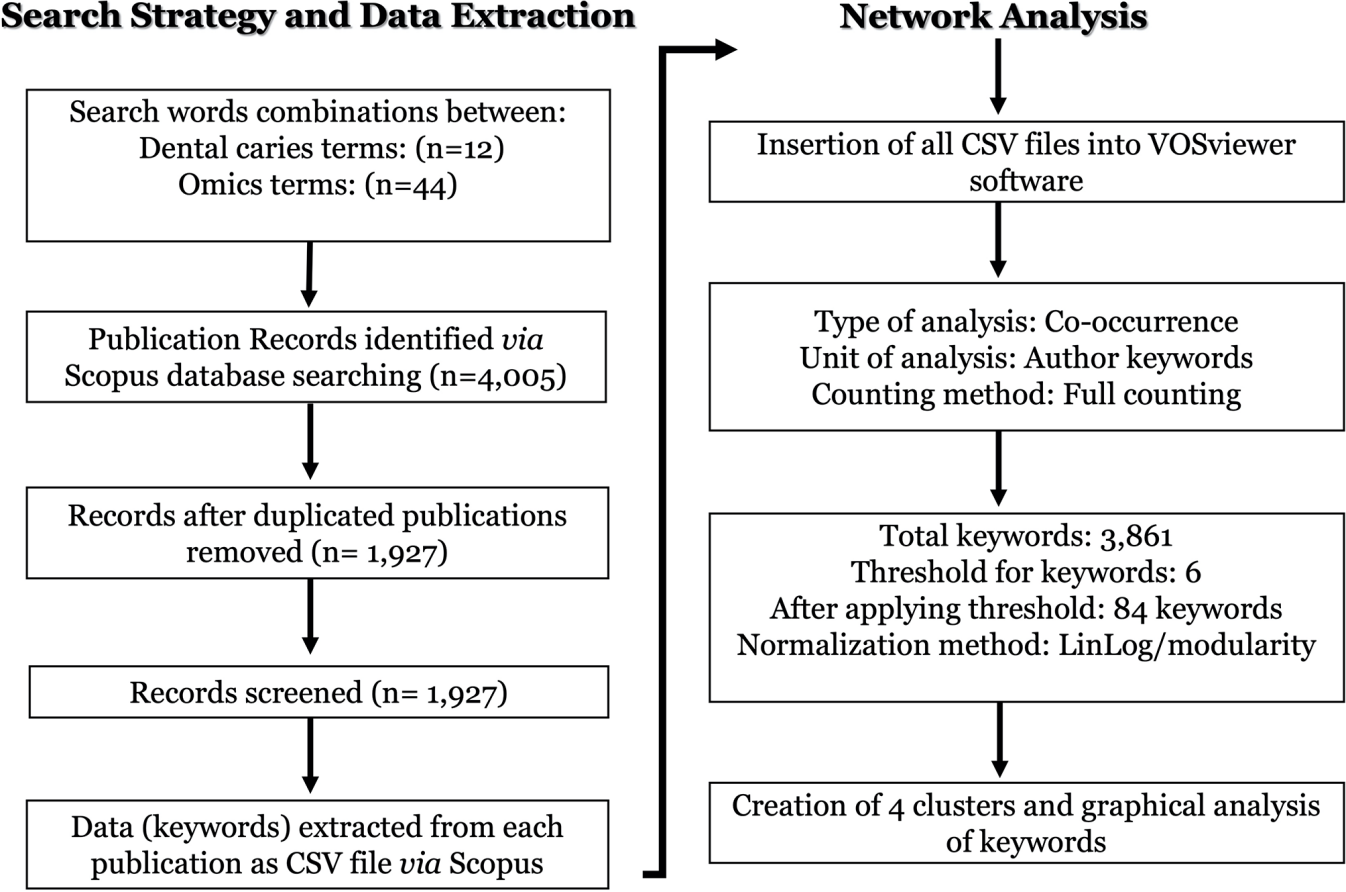
Flowchart of the search strategy, data extraction and networking analysis of the omics studies in dental caries research. The dental caries literature was screened on Novermebr1st, 2021 *via* Elsevier’s Scopus database. The search terms were identified to cover dental caries terms (n = 12) in combination with omics-related terms (n = 44) in either the article title and/or the abstract and/or the authors keywords as detailed in (**Table 1**). All search output files of identified publications (n = 4,005) were downloaded from the Scopus, concatenated, and duplicate entries were excluded. Deduplicated papers (n = 1,927) were imported to the Visualization of Similarities (VOSviewer) software for descriptive exploration and co-occurrence network analysis as described in the flowchart under the “network analysis”.

**Figure 4:**
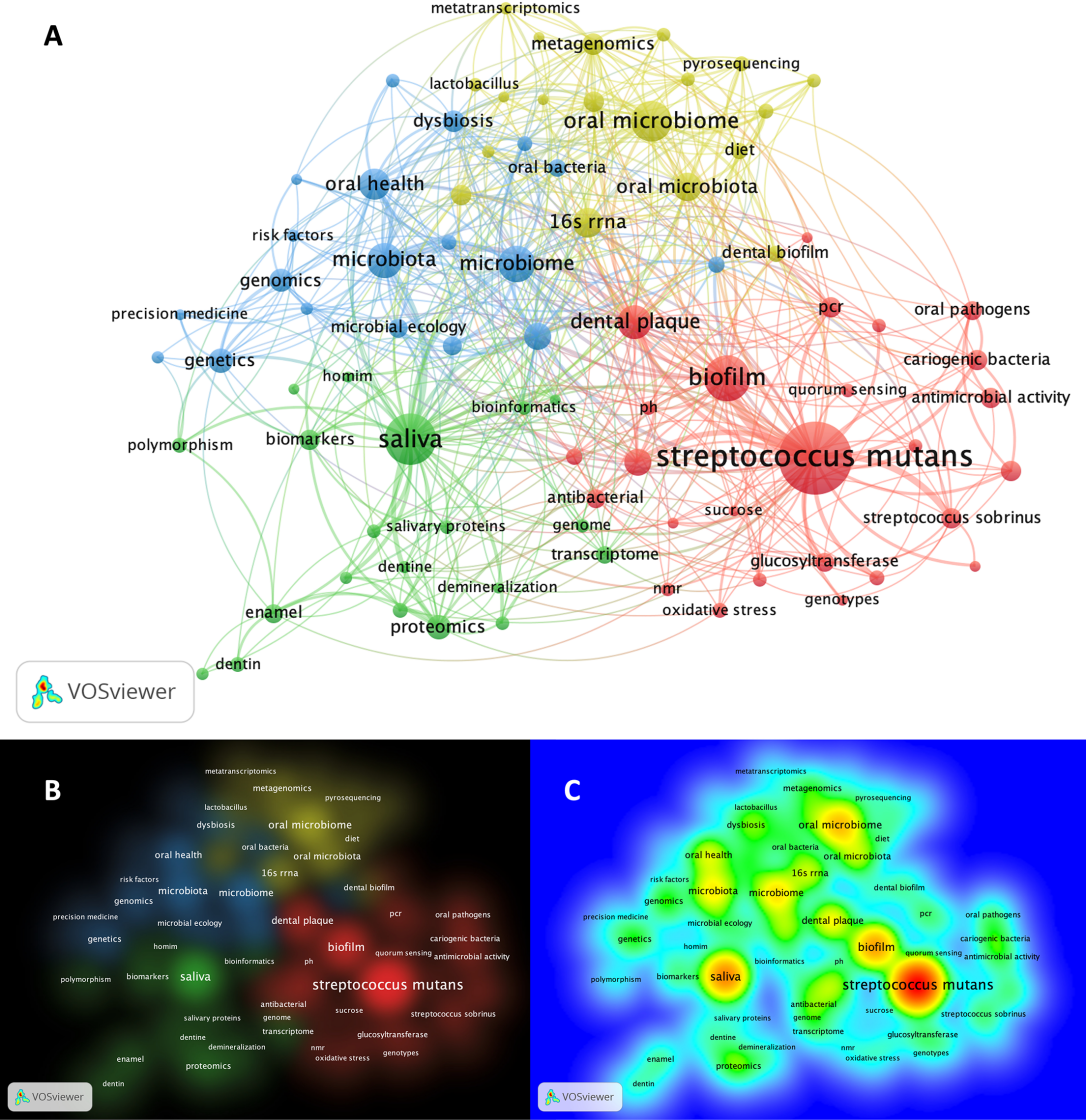
Bibliographic mapping of keywords associated with omic-related publications in dental caries research. **(A)** Mapping of keywords categorized cluster-based. The size of the nodes represents the frequency of keywords (larger nodes indicating higher frequency) and the distance between two terms, represent the strength of association between the terms (the smaller the distance, the higher the number of co-occurrences). Clusters are color-coded according to how relevant they appeared for the average number of times (threshold ≥ 6). **(B)** Pictograph representation of the keyword mapping showing the cluster density of the identified 4 clusters. Representative keywords form each clusters are listed as follow: *Cluster-1* (*red*): *streptococcus mutans*, oral *streptococci*, oral pathogens, *streptococcus sobrinus*, sucrose, pH, sever early childhood caries, quorum sensing, PCR, oxidative stress, acid tolerance, antibacterial, antimicrobial activity, biofilm, biofilm formation, cariogenic bacteria, dental plaque, genotype, genotypes, glucosyltransferase; *Cluster-2 (green)*: saliva, salivary proteins, proteome, proteomics, transcriptome, metabolome, mass spectrometry, genome, homim, homings, biomarkers, polymorphism, bioinformatics, hydroxyapatite, enamel, dentin, demineralization; *Cluster-3 (blue)*: genetics, genomics, risk factors, precision medicine, microbial ecology, epidemiology, microbiome, dysbiosis, microbiota, oral health; *Cluster-4 (yellow)*: 16S rRNA, 16S rRNA gene sequencing, high-throughput sequencing, metagenomics, metabolomics, metatranscriptomics, next generation sequencing, pyrosequencing, machine learning. **(C)** Pictograph representation of the keyword mapping categorized frequency-based showing the item density. The brighter the node, the more frequently used keyword. For example, *streptcoccus mutans*, saliva, biofilm and microbiome show the highest density (frequency), respectively where the precision medicine and metatranscriptomics show the lowest density within the setting of the shown keywords.

Matching publication records (n=4,005) were downloaded from the Scopus website and concatenated, then duplicate entries were excluded using a bash script https://github.com/DinaMoussa/DentalCaries. Deduplicated records (n=1,927) were imported to the Visualization of Similarities (VOSviewer) software for descriptive exploration and co-occurrence networking of keywords associated with these publications (**Figure 3**). In the VOSviewer software, we used the “*create*” button to choose “*create a map based on bibliographic data*”. Then, we chose “*read data from bibliographic database files*” and selected “*Scopus*” to load the deduplicated CSV file. For the type of analysis, unit of analysis, and counting method, we selected “*co-occurrence*”, “*author keywords*”, and “*full counting*” respectively. The output list of keywords was manually curated to focus the analysis on the four habitats in dental caries research - saliva, acquired enamel pellicle (AEP), supragingival dental plaque, and carious lesions (**Figure 1**). All subgingival habitats (gingival crevices, gingival crevicular fluid and subgingival dental plaque) were excluded from this study. We set a minimum number of occurrences of a keyword at n=6, and 84 keywords met the threshold out of a total of 3,861 keywords (**Figure 3**). A list of these 84 keywords was created to depict the occurrences and total link strength of each keyword. Nodes in the network were further clustered by applying the Lin/log modularity normalization method that uses agglomerative hierarchical clustering, a bottom-up approach (**Figure 3**) (Rathinam and Sankar, 2019). A multidimensional graphical analysis of keyword clustering was performed to generate a bibliographic map. The clusters are were color-coded according to how relevant they appeared for the average number of times. The node sizes represent the frequency of the keywords (larger nodes indicating higher frequency) and the distance between two terms represents the strength of association between the terms (the smaller the distance, the higher the number of co-occurrences) (**Figure 4A**). The same applies to the two other graphical representations for cluster and item density (**Figures 4B, C**) with the brighter nodes indicate more frequently used keywords.

### Network Remarks

The search results of dental caries- and omics-related terms are detailed for each combination in **Table 1** and the bibliographic map showed four main clusters (**Figure 4A**). The clusters were dominated by keywords related to *S. mutans* and biofilm (red), saliva (green), genomics and genetics (blue), and DNA/RNA meta-omics techniques (yellow) (**Figures 4A, B**). Detailed keywords for each cluster are presented in the bibliographic map and its caption (**Figure 4**). The largest node in the map, and thus the keyword appearing most frequently, was for *S. mutans*, denoting an extensive focus on *S. mutans* in dental caries research, followed by saliva, biofilm and oral microbiome (**Figures 4A, C**). The *S. mutans* node was closely connected with other nodes like the biofilm and dental plaque in the same cluster (red) but less connected with the clusters for saliva and meta-omic techniques (green and yellow); *S. mutans* was almost disconnected from the host genetics, genomics, and risk factor cluster (blue). The second largest dense node represented saliva-related research (green), and it showed a close connection with host-related studies (blue), followed by *S. mutans*-related studies (red); saliva-related research (green) was markedly less connected with the meta-omics techniques (yellow) (**Figures 4A, C**). The keywords with the lowest density (least frequent appearance) were “precision medicine” and “metatranscriptomics” (**Figure 4C**). However, clusters do not necessarily show all items in a map and keywords may overlap in this multidimensional framework.

In the coming sections, each layer in the biological flow of information from DNA to metabolites will be critically reviewed in correlation with the bibliographic network analysis, highlighting representative critical studies for the host and microbiota in separate and integrative arrangements.

## Meta-Omics for Microbial Community Profiling and Functional Characterization

### Amplicon Sequencing (16S rRNA Gene Sequencing)

Next-Generation Sequencing (NGS) technologies have revolutionized genomic research (Koboldt et al., 2013). Targeted (i.e., amplicon) NGS can retrieve millions of partial 16S rRNA gene sequences in one sequencing run (Metzker, 2010), enabling detection of several hundred oral bacterial species (Dewhirst et al., 2010; Ahn et al., 2011). The first 16S rRNA-based NGS study of oral microbial communities was conducted by Keijser et al. (2008) (**Figure 2**). The study tested the saliva and supragingival plaque obtained from healthy adults to define the commensal microflora. Since then, a flood of NGS studies have been conducted to identify caries-associated bacteria, circumventing the culturing obstacles (Paster and Dewhirst, 2000).

A surprising and paradigm-shifting finding from these studies was that the *S. mutans* is not the primary etiological taxa in dental caries; it could be a minority species or completely nonexistent in caries-active patients (Kleinberg, 2002; Aas et al., 2008; Tanner et al., 2011; Gross et al., 2012; Rudney et al., 2015; Moussa and Aparicio, 2020; Baker et al., 2021). Thus, research in the previous decade, based on the aforementioned misconception, had an extensive emphasis on *S. mutans* (**Figure 4**). Instead, NGS studies revealed that dental caries is a polymicrobial disease, and its etiology is beyond a single causative organism (Simon-Soro and Mira, 2015). Further, what in fact drives cariogenesis is the imbalanced shift in the microbial community structure, promoted by frequent carbohydrates and sugar consumption, rather than a single, specific community member such as *S. mutans* (Gross et al., 2012; Yang et al., 2012). Caries-associated microbial communities have been generally found to be less diverse than those of healthy counterparts. However, those dysbiotic communities display considerable inter-individual variability and caries associated taxa have not been consistent between studies (Tanner et al., 2011; Gross et al., 2012; Benítez-Páez et al., 2014).

Albeit 16S rRNA gene sequencing reveals a large degree of previously uncharacterized diversity, this approach has a number of limitations including its inability to detect bacterial taxa at the species-level resolution due to the short read lengths of the commonly used sequencing technique (Quince et al., 2009). Since many genera encompass both commensals and caries-inducing species, 16S rRNA gene sequencing does not allow precise interpretation of caries associations (Henssge et al., 2009). Other limitations include selective preference for some species due to PCR bias (Jo et al., 2016), chimeric reads and sequencing errors resulting in false inflation of operational taxonomic units (OTUs) (Quince et al., 2011), and incompleteness of reference databases causing failure in taxonomic assignment (Park and Won, 2018).

### Whole-Genome Shotgun Metagenomics

The whole-genome shotgun (WGS) metagenomic approach has emerged from progressive advancements in sequencing technologies, with potential to address 16S rRNA caveats such as biases inherent in PCR amplification of a single gene. Despite the sophisticated bioinformatics tools required to analyze the massive amounts of WGS data, the latter has provided unprecedented resolution and robust estimates of microbial community composition as well as enabled prediction of their potential functions in very diverse environments (Mullany et al., 2008; Jovel et al., 2016). The first metagenomic study of the supragingival dental plaque was conducted by Belda-Ferre et al., 2012 (**Figure 2**) (Belda-Ferre et al., 2012). Their description of the microbial community structure strongly favored the nonspecific plaque hypothesis for the development of dental caries (Marsh, 1994; Kleinberg, 2002). They stressed, in agreement with others, that *S. mutans* is not a significant contributor in these polymicrobial carious lesions. Moreover, they showed that *S. mutans* was sometimes completely absent, while other bacterial genera like *Lactobacillus, Actinomyces, Bifidobacterium, or Veillonella* comprised a significant proportion of communities (Aas et al., 2008). Also, they highlighted the important influence of other poorly studied bacteria such as *Propionibacterium* and *Atopobium* (Belda-Ferre et al., 2012). Other studies confirmed that there is no ‘caries-specific’ OTUs (Bolyen et al., 2019) and showed that microbiomes of healthy individuals were relatively conserved in contrast to the more variable community structures of caries individuals (Yang et al., 2012; Chen et al., 2015). Few other studies applied shotgun metagenomics for saliva or supragingival plaque, underscoring the necessity of species- and strain-level resolution for caries prognosis (Yang et al., 2012; Espinoza et al., 2018; He et al., 2018). These findings were consistent with the ecological hypothesis that suggested a mixed bacterial-ecological etiology, in which a shift in the microbial community structure is the key event underling the cariogenesis (Yang et al., 2012). By relating taxonomy to function, Belda-Ferre et al. (2012) were able to predict “who can do what”; one of the most powerful applications of the metagenomics approach. Remarkably, they found that individuals who had never suffered from dental caries showed an overrepresentation of certain functional categories, such as genes of antimicrobial peptides and quorum sensing. Likewise, clustering of functional assignments gave similar patterns in diseased individuals, indicating that a similar set of functions were encoded in their metagenomes. Accordingly, the relative proportion of these functional categories can provide important insights into the ecology of each ecosystem and the potential role of the corresponding microbiota (Belda-Ferre et al., 2012). Nonetheless, it remains that functional omics that are closer to phenotyping are necessary to describe the disease trajectory rather than genome-based predictions, especially with the dynamic nature of dental caries (Franzosa et al., 2015; Heinken and Thiele, 2015; Schaubeck and Haller, 2015; Nowicki et al., 2018). Moreover, functional omics could provide promising high-resolution pictures of bacteria-host interactions (Franzosa et al., 2015; Franzosa et al., 2015) as described in the next sections.

### Metatranscriptomics

NGS technologies have enabled a new way to assess the gene expression (transcription activity) of bacteria that is referred to as RNA-seq (Mader et al., 2011; Pinto et al., 2011). A metatranscriptome provides a snapshot of the genes expressed in a microbial community at a given moment and under specific conditions *via* analyzing its mRNA. Therefore, unlike the metagenome, the metatranscriptome reveals the composition and active functions of a microbial community (Bashiardes et al., 2016). Metatranscriptomic studies in dental caries research are few and are marginalized as shown in the bibliographic analysis (**Table 1** and **Figure 4**) Furthermore, there is a significant deficiency in metatranscriptomic studies under healthy oral conditions that can be used as a reference (Solbiati and Frias-Lopez, 2018).

Benítez-Páez et al., 2014 presented the first metatranscriptomic study for human dental plaque (**Figure 2**) (Benítez-Páez et al., 2014). Their data showed not only that microbiota are individual-specific but also that microbiota considerably change their activity during biofilm formation. The expression of genes related to translation machinery was higher in early stage biofilms, whereas more specialized genes were expressed in mature biofilms. Particularly in early stages, they found that the predominantly upregulated genes were involved in the metabolism of carbohydrates and amino acids, whereas in the late stages, the upregulated genes were related to quorum sensing response; this indicates that the community is adapting as it develops. Intriguingly, more than 80% of active bacteria were affiliated to only three genera (*Actinomyces*, *Corynebacterium*, and *Rothia*), which infers that the mere existence of certain species does not necessarily prove their active contribution in the cariogenesis process (Benítez-Páez et al., 2014). The same conclusion was also reached by others (Solbiati and Frias-Lopez, 2018). Similarly, another study concluded that despite the high inter-patient variability in the microbial community composition, caries-associated communities display conserved functional categories and transcripts which are produced by a limited number of patient-specific cohorts of microbe (Peterson et al., 2014). Based on the community-wide expression profile, the most active taxa found in dental plaque samples from dental caries patients were *S. sanguinis* (16% of the transcripts), followed by *Streptococcus mitis* (10%), *Veillonella parvula* (9%), *Capnocytophaga* sp. (9%), *Streptococcus oralis* (8%), *Streptococcus* spp. (7%), *Gemella haemolysans* (5%), *Streptococcus gordonii* (4%), and *Neisseria* sp. (3%) (Peterson et al., 2013; Peterson et al., 2014). The distinct functional patterns observed were associated with oxidative stress and high expression of proteins that metabolize super-oxides and peroxides. Distinctively, sugar metabolism was identified as a major element that distinguishes health and carious conditions (May et al., 2016; Tanner et al., 2017; Solbiati and Frias-Lopez, 2018). Although mRNA is a closer approximation of metabolism than genome-based predictions, it is not an exact prediction of metabolic activity. Therefore, the research paradigm has recently focused on studying the translational bacterial proteome and/or metabolome, i.e. the closest links to the phenotype (**Figure 1**), to determine the origin of proteins and metabolites produced by the microbiome under different conditions (Kohler et al., 2016; Jacob et al., 2017).

### Metaproteomics

Metaproteomics characterizes proteins expressed by both microbial communities and their hosts which reveal insights into the molecular functions conferred by these communities as well as the host-microbe interactions (Jagtap et al., 2015). In this section, we focus on metaproteomics for the microbial side and defer discussion of the host side to the host-related omics section.

Like metatranscriptomics, metaproteomics also analyzes community microbial expression but instead analyzes at the proteome level to decipher the function of microbial communities or to identify microbial functional networks (Hettich et al., 2013). Metaproteomic study of whole saliva has been utilized for diagnostic purposes since it includes a complex balance of secretions from salivary glands, oral mucosa, epithelial cells, serum, crevicular fluid, bacteria and food debris (Siqueira and Dawes, 2011). Moreover, rapid advances in Mass spectrometry (MS) based proteomic profiling and bioinformatic tools in the last two decades have opened new horizons for functional characterization and biomarker discovery (Bostanci and Bao, 2017). Nonetheless, compared to conventional proteomics, metaproteomics presents unique data analysis challenges since it requires the use of large protein databases derived from hundreds or thousands of organisms as well as requires several processing steps. Furthermore, the number of bacterial proteins identified in saliva is scarce, which necessitates the multidimensional approaches used in saliva fractionation (Vitorino et al., 2012). These challenges limit metaproteomics investigations for many researchers (Jagtap et al., 2015). In an early attempt by Rudney et al., 2010, they applied a combined three-dimensional metaproteomics approach that identified 139 proteins from 34 different salivary microbiota (Rudney et al., 2010). In 2012, Jagtap et al. presented the first deep metaproteomics of human salivary supernatant (**Figure 2**) (Jagtap et al., 2012), in which the taxonomic diversity observed was consistent with metagenomic studies of saliva (Keijser et al., 2008; Nasidze et al., 2009; Bik et al., 2010). Their metaproteomic catalog provided a baseline for future studies examining shifts in microbial diversity and protein activities potentially associated with oral diseases (Jagtap et al., 2012). Since it was now evident that taxonomy alone could not explain dental caries, Belda-Ferre et al. who conducted the first metagenomics of the oral biofilm, followed up with the first metaproteomic study (**Figure 2**) aiming to relate bacterial composition and functions to caries incidence *via* two different analytical platforms (Belda-Ferre et al., 2012; Belda-Ferre et al., 2015). They managed to quantify a total of 7771 bacterial and 853 human proteins providing the first available protein repertoire of clinical human dental plaque. Potential biomarkers abundantly present in disease state were the glucose phosphotransferase system, copper-containing nitrite reductase, and stress response protein CspD. These pioneering findings were an initial approach to identify functional biomarkers for early diagnosis of caries-prone individuals (Belda-Ferre et al., 2015).

One major concern regarding metaproteomics of the complex systems consisting of hundreds of different organisms is the domination by a few distinctive proteotypic peptides because they are highly responsive to the analysis (Belstrøm et al., 2016). Domination by these specific peptides makes correlation between experimental observations and what is actually occurring in the natural complex system questionable, inconsistent and unquantifiable. Additionally, normalization factors, used to calculate bacterial protein abundance ratios, are generally quite modest as they are usually less than 20% of the total observed signal for any given proteome (Belstrøm et al., 2016; Grassl et al., 2016). For instance, it was promising that Rudeny et al., 2015 identified conserved protein relative abundance patterns across dysbiotic and taxonomically diverse oral biofilms that could be used to detect at-risk tooth surfaces before the development of overt carious lesions (Rudney et al., 2015). Conversely, Belstrøm et al. (2016), who presented the first characterization of the salivary metaproteome, found that the bacterial part of the metaproteome is unsuitable as a biomarker for caries; however, they found a potential set of host-specific biomarkers (Belstrøm et al., 2016). In a recent state-of-art shotgun proteomic study, more than 5500 proteins were identified from whole saliva of healthy individuals, the largest number of human proteins identified in a bodily fluid (Grassl et al., 2016). The authors concluded that shotgun proteomics can determine 50 bacterial genera in saliva that show close agreement with next generation sequencing data from the Human Microbiome Project (2012). Nevertheless, the coverage of bacterial proteins revealed that they have merely scratched the surface of the oral bacterial proteome (Grassl et al., 2016). Collectively, the difficulties involved in performing metaproteomics, such as inconsistent reproducibly and expense, have been an issue compared to what can be more easily achieved *via* metatranscriptomics at the same level of coverage and quantitative reliability (Kuboniwa et al., 2009; Kuboniwa et al., 2012). Although metaproteomics is a powerful tool for studying the composition, abundance, and modular organization of proteins in oral microbial communities that govern the phenotype, the aforementioned difficulties and concerns may explain why there are only a handful of studies published in dental caries research (**Table 1**) (Bostanci et al., 2021). Still, proteomics is uniquely positioned to analyze the interplay between microbial protein expression and the host response which will be addressed under the host-microbe interaction section.

### Metabolomics

The metabolome is the closest link to the phenotype that reflects all the information expressed and modulated by all other molecular omic-layers along the biological hierarchy (**Figure 1**) (Nyvad et al., 2013). Metabolic profiling can give an instantaneous snapshot of the physiology of an ecosystem, i.e. reveal current activity, rather than identifying the state conducive to a particular condition (Bostanci et al., 2021). Additionally, metabolic profiles can offer deep insights into the impact of lifestyle and dietary factors at different stages of a disease (Bostanci et al., 2021). Since metabolomics provides a direct functional readout of the current physiological state, it is at the forefront of personalized diagnosis and therapy (Kohler et al., 2016).

The National Institute of Health (NIH) Human Microbiome Project consortium (NIH HMP Working Group et al., 2009) validated the existence of stable metabolic pathways for supragingival plaque across a cohort of individuals, despite a large variation in the taxonomic structure (NIH HMP Working Group et al., 2009; The Human Microbiome Project Consortium, 2012). This finding opened up the prospect of searching for conserved functional biomarkers for dental caries that are less dependent on taxonomy. A small number of studies pursued this approach to reveal how metabolic pathways are altered in cariogenic conditions.

Identifying and quantifying metabolites is typically carried out using a combination of chromatography techniques (liquid chromatography, LC, and gas chromatography, GC) and detection methods, such as MS and nuclear magnetic resonance (NMR). The strengths and weaknesses for each approach, as well as the technical specifics, are systematically addressed by Aldridge and Rhee (2014). The Takahashi group presented a number of *in-vitro* and *in-vivo* studies aiming to explore a metabolite signature for caries activity in the supragingival plaque biofilm using an MS-based method (**Figure 2**) (Takahashi et al., 2012; Takahashi, 2015). They detected that environmental acidification is the main determinant of the phenotypic and genotypic changes that occur in the microflora of the caries environment (Takahashi and Nyvad, 2011). Therefore, they focused on the central carbon metabolism (Embden- Meyerhof-Parnas “EMP” pathway, pentose phosphate pathway, and Krebs cycle) since the organic acids as metabolites have shown a direct relation to cariogenesis. Several consistently upregulated metabolites have been found along their targeted pathways under *in-vivo* cariogenic conditions (Takahashi et al., 2010). Interestingly, they observed the same findings upon *in-vitro* testing of *Streptococcus*, *Actinomyce*s, and *Lactobacillus* under similar conditions (Takahashi and Yamada, 1999; Takahashi et al., 2010). Collectively, they concluded that although oral biofilm consists of a tremendous number of microorganisms, they function as a one organism in dysbiotic environments (Takahashi et al., 2012).

Other studies investigated metabolites in saliva using an NMR-based method in children with and without dental caries and after caries treatment (Fidalgo et al., 2013; Fidalgo et al., 2015). Their results demonstrated a distinction between the saliva derived from children with and without caries. Particularly, organic acids (acetate, propionate, fatty acid, butyrate and saccharides) were associated with disease activity and significant reduction was noticed after caries treatment. Likewise, they identified similar salivary metabolite profiles for healthy subjects in spite of the differences in their oral hygiene habits, socioeconomic status, and food intake.

Since there is a consensus on acidity as a major driving factor for cariogenesis, researchers sought to explore the contribution of major pathways of amino acids metabolism to the pH homeostasis. Washio et al., 2016 studied how amino acids are metabolized in the supragingival plaque and measured the amounts of ammonia and amino acid-related metabolites along with the carbohydrate metabolism. Their metabolome profile revealed that amino acids are degraded through various metabolic pathways. However, the specific pathways responsible for alkalization, which can counteract cariogenic acidification, were still not fully clarified (Washio et al., 2016).

Detailed metabolome profiling of the AEP is quite lacking; however, Schulz et al. have recently characterized the 10-min AEP in children with different caries activity in comparison to saliva (Schulz et al., 2020). No significant difference was found among the groups for the tested metabolites (amino acids, organic acids, fatty acids and carbohydrates) and no single typical metabolite profile was thus found to represent caries. The authors believed that metabolites in the AEP seem to be independent of caries status and are not suitable for caries diagnosis (Schulz et al., 2020). Similarly, another study focused on the pellicle’s enzyme activity did not find significant differences related to caries activity, concluding that metabolites in short-term pellicles are unlikely to be identified as markers for caries activity (Hertel et al., 2019).

Valuably, metabolomics complements the information provided by the other aforementioned omics data layers and describes the complex metabolic interactions between the host and microbial partners (Moco et al., 2013; Kim et al., 2016). Nevertheless, one challenging aspect of this approach is to determine whether a given metabolite was generated by the host or by the microbiome. Hence, in order to determine which genes, enzymes, and/or pathways are associated with a specific metabolite, the results obtained from a metabolomic study must be combined with other omic profiles. This underlines the need for new approaches of integrated omics, as discussed in the coming section (Aguiar-Pulido et al., 2016).

### Integrated Meta-Omics

Standard analyses of omic datasets focus on either the community structure or functions. Elucidating the complex network of interactions between the constituent entities continues to be a significant challenge. In the complex oral ecosystem, understanding the disease nature and dynamics requires a multi-level analysis that includes taxonomic assessment (composition), potential functions (metagenome), active functions (metatranscriptome), and encoded functions (metaproteome and metabolome) (Duran-Pinedo and Frias-Lopez, 2015). Although each approach provides valuable information separately, their integrated downstream analysis is significantly more valuable than the sum of its parts in gaining a comprehensive understanding of the disease nature and dynamics (Aguiar-Pulido et al., 2016). Therefore, a complete understanding of the caries onset and progression cannot be accomplished by applying a single technique, but rather by combining at least two or more (Gomez and Nelson, 2017). In spite of these potential benefits, such an approach has been seldomly implemented in dental caries research (**Figure 4**).

Studies in various biomedical fields have indicated that integrating metagenomics and metatranscriptomics has the potential to reveal over-or under-expression of particular functions and the activities of specific organisms (Mason et al., 2012; Duran-Pinedo et al., 2014; Franzosa et al., 2014). Other studies suggest that metagenomics and metabolomics integration can provide deep insight into the whole ecosystem based on differential expression of specific metabolites that impact the health of the host environment (Turnbaugh and Gordon, 2008; Wang et al., 2011; Weir et al., 2013). Integrating all three omic data - metagenomics, metatranscriptomics, and metabolomics - was furthermore recommended as a mean of providing a complete picture from genes to the phenotype (Muller et al., 2013; Narayanasamy et al., 2015). A practical approach is to perform a separate analysis and add an extra integrative step within the downstream analysis (Segata et al., 2013; Aguiar-Pulido et al., 2016). These examples highlight the need for integrative analyses that deal with multi-omic datasets to investigate the different levels of complexities inherent in biological systems. We will highlight some studies that used different integrative approaches to study the dynamics of dental caries in different habitats (**Figure 2**).

Benítez-Páez et al., 2014 presented the first example of combining two metatranscriptomic datasets and used two different sequencing approaches, low coverage long reads and high-coverage short-reads, for compositional and functional analysis of the microbiota (**Figure 2**) (Benítez-Páez et al., 2014). Their high-coverage approach revealed that microbial communities are individual-specific and no bacterial species was detected as a key player at any time during biofilm formation; however, they did identify gene expression patterns that were distinctive for early and mature oral biofilms. For example, genes involved in competence, quorum sensing, mutacin production, and DNA uptake were over-expressed in late biofilms. Additionally, the long reads showed individual-specific changes before and after carbohydrate-rich meals, which narrowed down the list of active organisms responsible for acid production and, possibly, the eventual dental caries. Yet, they advised of the need for further studies to characterize the identified genes, as more than 70% of the genetic information compiled from this oral metatranscriptome had no functional assignment (Benítez-Páez et al., 2014). One year later, Edlund et al., 2015, presented the first integrative metatranscriptomic and metabolomic analysis of a diverse oral plaque community *in-vitro* model (Edlund et al., 2015). Their aim was to move beyond single species applications toward a complex multispecies microbial model to identify the active key players and their concomitant metabolic activities. Distinct signatures in the metabolite profiles and corresponding transcriptional profiles were observed, especially for pH neutralizing pathways. They concluded that their model would serve as a baseline for defining healthy and disease-like states from the metabolome and transcriptome of supragingival plaque samples.

For the human saliva habitat, the first study to perform simultaneous metagenomic and metatranscriptomic characterization of the salivary microbiota was conducted by Belstrøm et al., 2017 (**Figure 2**) (Belstrøm et al., 2017). They analyzed the microbial diversity and differentially expressed microbial genes in patients with periodontitis and dental caries that could be used as proxy biomarkers of oral health and disease. On the one hand, aligning with other studies using metagenomic data extracted from supragingival biofilms (Belda-Ferre et al., 2012; Benítez-Páez et al., 2014), they observed major differences in bacterial metagenomic profiles in saliva. On the other hand, they observed consistent differential salivary expression of specific metabolic genes up-regulating carbohydrate metabolism and down-regulating lipid-metabolism in dental caries patients (Belstrøm et al., 2017).

## Host-Omics for Genetic-Profiling and Functional Characterization

It should be taken into account that the susceptibility for dental caries among people varies even when they exhibit the same lifestyle habits (Krasse, 2001; Rosier et al., 2018). This explains why individuals with similar behavioral risks (i.e. tooth brushing frequency or dietary habits) can have different caries risk and/or caries activity (Bretz et al., 2003). The importance of the host-dependent milieu in selection of the colonizing bacterial species has been validated. Moreover, the virulence of certain species could differ substantially among different ethnic individuals (Rosier et al., 2014). The periodontium research has stepped forward by introducing the polymicrobial synergy and dysbiosis (PSD) model that entails different gene combinations of dysbiotic microbiota and susceptible hosts for transition to periodontitis (Hajishengallis and Lamont, 2012; Hajishengallis and Lamont, 2014). However, thus far, there is no similar model for dental caries that encompasses possible host factors and their interactions with microbial communities in the maintenance of health or the shift to dental caries disease. Such disconnection is discernibly reflected in the research that has been conducted; as portrayed in the bibliographic mapping, the host genetics and genomics are almost un-integrated with the microbiome research (**Figure 4**). Thus, an all-inclusive hypothesis is critically needed, but this is only possible with sufficient knowledge of the complex relationships between the oral microbiome and the host’s genetic background.

### Host Genomics and Transcriptomics

Since the mid-1900s, the genetic linkage in caries etiology has been investigated by a various approaches (**Figure 2**) and heritability has been estimated to be 20-60% (Boraas et al., 1988; Bretz et al., 2005; Wang et al., 2010; Shaffer et al., 2012; Shaffer et al., 2015; Morrison et al., 2016). To study the genetics of a complex trait such as caries, candidate gene studies and genome-wide studies are the two main study models usually applied. On the one hand, candidate gene studies test specific hypotheses regarding association between specific genes and the disease. Candidate gene studies focused mainly on genes related to enamel development, immune response, saliva composition, taste preferences and eating habits (Wright, 2010; Vieira et al., 2014). Identified associations have not always been consistently replicated and this might be attributed to population heterogeneity and/or statistical power issues (Nibali et al., 2017). On the other hand, genome-wide studies test the disease association with a large number of DNA variants throughout the genome. While gene-mapping of dental caries is still in its childhood, genome-wide studies have begun to identify regions in the genome that are likely to harbor caries risk genes.

Vieira et al., 2008 presented the first genome-wide linkage study for dental caries in large families (**Figure 2**) and suggested five loci linked to caries experience (Vieira et al., 2008). Marazita’s group pioneered in applying Genome-Wide Association Studies (GWAS) to understand the specific contributions of genetic and environmental factors in the etiology of dental caries (**Figure 2**) (Shaffer et al., 2011; Wang et al., 2012). Several biologically plausible putative genes and loci were found to be relevant to tooth development, saliva composition, and immune defense (Wang et al., 2013). However, very little overlap was found across different genome-wide linkage and association studies (Vieira et al., 2014). Accordingly, examining the joint effects of multiple risk loci, such as the gene set enrichment approach, has garnered more attention to identifying the functional relationships between caries-associated genetic factors (Wang et al., 2013). All studies investigating candidate genes as well as those adopting genome-wide approaches until 2014 are extensively reviewed by Vieira et al. (2014).

Until 2017, nominated genes could not be confirmed as no genetic variants had clearly been associated, as reported in the meta-analysis of several genome-wide studies for dental caries (Nibali et al., 2017). The authors underscored the small sample size of the reviewed studies and suggested further studies with larger sample size are warranted for a complex trait such as caries. In 2018, a large-scale consortium-based GWAS (with over 19,000 individuals) examined the genetic loci associated with dental caries in primary and permanent dentition (Haworth et al., 2018). The consortium-wide estimated heritability of caries was surprisingly low, at 1-6%, compared to corresponding within-study estimates or previously published estimates. The authors attributed these unexpected results to several factors in their study design, prioritizing two main factors. First, the analysis method was based on single-nucleotide polymorphisms (SNPs) association, which consistently underestimates heritability of complex traits. Second, the phenotypic definitions used in this study did not depict the caries extent or severity, which might have contributed to the low statistical power of analysis (Haworth et al., 2018; Docherty et al., 2016).

Govil et al., 2018 conducted the first study applying the genome-wide multipoint linkage approach to make full use of familial inheritance to explore the genetic etiology of caries. Promisingly, the linkage findings nominated genes on six chromosomes as having potential involvement in caries etiology, either directly or *via* an indirect contribution in human immune and host defense response, blood glucose levels, and secretory function of the salivary glands (Govil et al., 2018). In another approach, the Vieira group presented the first study to separate dental caries into more discrete sub-phenotypes, defined from longitudinal data, to better aid in gene identification studies. They found significant associations between a number of SNPs and their resulting caries phenotypes; however, there was uncertainty regarding the direct causality of these genes to dental caries. Accordingly, they strongly recommend further studies to identify the potential mechanisms and roles these genes play in dental caries (Weber et al., 2018). In a recent comprehensive collaborative work, forty-seven novel and conditionally-independent risk loci for dental caries were identified, showing that the heritability of dental caries is enriched for conserved genomic regions (Shungin et al., 2019). This study leveraged detailed clinical measures in combination with genetically validated proxy phenotypes to identify the risk loci for dental caries (Shungin et al., 2019). To that end, characterizing both the functional biology at these loci and the interaction between putative caries risk conferring genes and the microbiome need to be investigated.

In contrast to GWAS, transcriptome-wide association studies (TWAS) is a powerful approach that can detect novel disease loci or ascertain the susceptibility genes at known disease loci (Barfield et al., 2018). The TWAS approach was recently developed by Gusev et al. to integrate tissue expression with variants found to be associated with the studied phenotype in a GWAS (Gusev et al., 2016). Tong et al., 2020 were the first to conduct an integrative analysis of TWAS and mRNA expression profiling of dental caries to identify commonly associated genes and gene ontology (GO) terms (**Figure 2**) (Tong et al., 2020). Six common candidate genes were observed including CTSC, STAB1, and NCF2. Interestingly, several identified CTSC mutations were previously found to be associated with severe dental caries and periodontitis (Wei et al., 2020); this might explain the correlation between dental caries and periodontal diseases (Mattila et al., 2010; Durand et al., 2019). Also, STAB1 and NCF2 were found to be involved in the activation of macrophages and neutrophils, respectively. Further, five common GO terms were identified; among these are three critical terms for positive regulation of cell migration, response to hypoxia, and extracellular exosome that are associated with advanced stages of the disease and the apoptosis of human dental pulp cells. This method is novel and has had very limited applications in the field; therefore, further research is needed to verify these findings and reveal potential functions and genetic mechanisms of the identified genes in cariogenesis.

### Host Proteomics

It was shown that the composition, function, and properties of saliva, which are key elements in host defense, play essential roles in the maintenance of the surface integrity of dental hard tissues (Siqueira et al., 2007; Gao et al., 2016). These protective roles include the pellicle layer that is formed by proteins, carbohydrates, and lipids spontaneously adsorbing onto enamel surfaces (Siqueira et al., 2012). Dawes et al., 1963 was the first to describe this proteinaceous layer and introduce the term acquired enamel pellicle (AEP) (Dawes CT, 1963), which has a close compositional relationship to saliva (Siqueira et al., 2007). Moreover, the biological composition of saliva has been found to reflect individual oral health status (Almstahl and Wikstrom, 1999; Dawes et al., 2015). Therefore, constituents of both AEP and saliva can be provoked by different oral conditions including dental caries (Siqueira et al., 2007; Vukosavljevic et al., 2011).

In 2007, Siqueira et al. analyzed the global proteome of the human AEP (**Figure 2**). A total of 130 proteins were identified and categorized into three groups: proteins that have the ability to bind calcium ions (18%), phosphate ions (15%), or other proteins (28%) (Siqueira et al., 2007). Recent advances in protein fragmentation methods and MS technologies enabled further analysis of proteomic profiles from the AEP, resulting in identification of up to 363 various peptides and proteins (Zimmerman et al., 2013).

Regarding saliva, the salivary proteome has also been characterized and 3,000 proteins were identified. These proteins are predominated by amylase, carbonic anhydrase, mucins, cystatins, proline-rich proteins, histatins, and statherin (Hu et al., 2007; Denny et al., 2008; Bandhakavi et al., 2009).

Despite the comprehensive proteomic profiling of the AEP and saliva, only few inconsistent attempts have studied the suitability of salivary protein concentrations to serve as caries biomarkers (Laputkova et al., 2018). Correspondingly in our bibliographic network mapping, their nodes appeared small and scattered in the green cluster, indicating their low frequency and dispersion (**Figure 4**). This dispersed pattern might be explained by difficulties encountered in this type of analysis. For example, salivary proteins display considerable redundancy in their functions, some proteins appear to modulate each other’s functions, and/or others form heterotypic complexes that behave differently from their component parts (Moussa and Siqueira, 2021). Additionally, variables such as sex, age, diet, circadian rhythm, individual variability, and sample stability might influence data retrieved from proteomic analysis (Vitorino R et al., 2009; Amado et al., 2013). For instance, proteomic analysis of saliva performed by Rudeny et al. concluded that levels of statherin and truncated cystatin S may be potential risk indicators for caries development (Rudney et al., 2009). Despite the unproven antimicrobial functions of these protein, their findings could be rationalized if one assumes that higher levels of statherin are associated with a higher rate of remineralization in caries status, given statherin’s significant role in mineralization (Hay et al., 1984). Additionally, statherin has been observed to inversely correlate with the supragingival biofilm (Rudney et al., 2009) and inhibit bacterial adhesion to other salivary proteins (Shimotoyodome et al., 2006). However, these findings contradicted two other studies that found statherin and cystatin S levels were higher in the caries-free state (Vitorino et al., 2005; Vitorino et al., 2006). Similarly, Al-Tarawneh et al.’s review concluded that, until 2009, salivary biomarkers were inconsistently identified for most of the dental caries studies using MS proteomics (Al-Tarawneh et al., 2011). In 2013, Martins et al., in agreement with Al-Tarawneh et al., concluded that no sufficient evidence exists to establish salivary proteins as a biomarker for this disease (Martins et al., 2013).

Newer proteomic studies were designed to characterize both human and bacterial proteins. In 2015, Belda-Ferre presented the first protein repertoire of human dental plaque, characterizing both the human and bacterial parts of the oral biofilm (**Figure 2**) (Belda-Ferre et al., 2015). They quantified individual peptides in healthy and caries-bearing individuals and identified a total of 853 proteins in 17 individuals. Healthy individuals showed significantly higher amounts of L-lactate dehydrogenase and the arginine deiminase system, which are both linked to pH buffering. Other proteins found to be significantly higher in healthy individuals, are involved in exopolysaccharide synthesis, iron metabolism and immune response (Belda-Ferre et al., 2015).

In 2016, the Belstrøm group presented the first study to characterize both the human and bacterial parts of the salivary metaproteome in patients with dental caries (**Figure 2**). The bacterial part of the metaproteome seemed to be inadequate as biomarkers for caries. Conversely, the authors found a set of overexpressed proteins related to the complement system and inflammation, with potential to be used as host-specific biomarkers. However, their cross-sectional study design did not allow them to address the causality of this observation (Belstrøm et al., 2016). It has been noted that large-scale longitudinal studies, larger sample size (largest sample size reported was 41), several replicates per sample, and standardization of sample collection/treatment protocol are warranted to discover biomarkers of oral health and disease (Al-Tarawneh et al., 2011; Belstrøm et al., 2016). Furthermore, since identification of specific disease biomarkers remains a challenge, integrating the expression profiles of the host with the microbiome patterns is a direction that should be pursued in future studies.

## Host-Microbiome Interaction

The oral cavity harbors one of the most complex polymicrobial communities, encompassing interactions with several host factors (e.g., tooth anatomy and immune response) and environmental factors (e.g., food and brushing habits) that eventually control the health state as well as cariogenesis (Lamont et al., 2018). Typically, homeostasis, a balanced-relationship, exists between the host and the microbial community, even in the presence of pathogens that are an inherent part of this ecosystem (Kamada et al., 2013). Nevertheless, under particular conditions where perturbations exceed thresholds, homeostasis is compromised and results in imbalanced dysbiotic conditions that trigger site-specific carious lesions (Lamont and Hajishengallis, 2015). Thus, focusing on one component of this dynamic milieu will not unravel the causality and prognosis of this disease in its various stages.

Lamont et al., 2018, published a review article to discuss the dynamic microbial communities and their host interactions in dental caries and periodontal diseases (Lamont et al., 2018). The authors discussed how alterations in the host’s immune competence, saliva, diet, composition of the extracellular polymeric matrix, complex signaling, and cross-feeding interactions can affect the meta-transcriptional landscape. For example, a number of studies have illustrated that dentin caries is associated with elevation of host signaling molecules and certain cytokines (Hahn et al., 2000; Artese et al., 2002; McLachlan et al., 2004; Adachi et al., 2007; Horst et al., 2011). Nevertheless, co-occurrence omics studies for host-microbe markers in dental caries are very deficient. Lamont et al. advised that integrating omics data with microorganism phenotype-pathogenicity association, together with complementary polymicrobial models, is an influential strategy with potential to move beyond correlations and ascertain the actual disease-causation (Lamont et al., 2018).

The Stiesch group presented a study model for host–microbe interaction on a related topic of inflamed mucosa in the peri-implant diseases (Ingendoh-Tsakmakidis et al., 2019; Mikolai et al., 2020). Briefly, they developed a novel, simulating *in-vitro* three-dimensional model comprised of three components - organotypic oral mucosa, implant material, and oral biofilm - with structural assembly close to the native situation to investigate the early host-microbe interaction (Ingendoh-Tsakmakidis et al., 2019). The model enabled the qualitative and quantitative characterization of biofilms and histological characterization of the peri-implant mucosa. Moreover, the model enabled molecular identification of differentially expressed genes and monitoring of the markers of immune response, including cytokines and human β- Defensins. Disruption of homeostasis was accompanied by an enhanced immune response with upregulation of inflammatory-related genes and increased cytokine secretion. This model of host-microbe interaction at the peri-implant site may pinpoint changes associated with disease onset and provide the basis to improve strategies for prevention (Ingendoh-Tsakmakidis et al., 2019; Mikolai et al., 2020).

Similarly*, in-vivo* murine models were used to decipher microbial-host interactions for the gut microbiota, as described by Kovatcheva- Datchary et al. (Kovatcheva-Datchary et al., 2019). They developed a mouse model colonized with a simplified microbiota, containing ten known bacterial strains from the human gut microbiome. The mice were fed different diets to observe the changes in bacteria abundance with diet changes. Concurrently, they annotated the altered genes associated with metabolism variations *via* RNA sequencing. Additionally, circulating metabolites can be detected *via* metabolomic analysis of the mice plasma (Kovatcheva-Datchary et al., 2019). These aforementioned *in-vitro* and *in-vivo* models are examples of study designs that could move forward the much-needed investigations of host-microbe interactions associated with the disease onset and progression in dental caries research.

This year, Moussa et al., 2022 introduced the first *ex-vivo* model with integrated multi-omics of the early onset of dental caries; it focused on determining the disease etiology at an otherwise clinically undiagnosable stage (**Figure 2**) (Moussa et al., 2022). They developed an innovative longitudinal *ex-vivo* model combined with the non-invasive multiphoton second harmonic generation bioimaging to spot the very early signs of dental caries in human teeth using supragingival plaque microcosms. Their model enabled detection of the exact onset of the disease as well as simultaneous characterization of the associated microbiome changes and functional phenotypes using integrative 16S metagenomics and targeted metabolomics. Further, they elucidated concomitant co-occurrences between microbial and metabolic biomarkers. Specifically, five differentiating metabolites were recognized - Lactate, Pyruvate, Dihydroxyacetone phosphate, Glyceraldehyde 3-phosphate (upregulated) and Fumarate (downregulated) - which co-occurred with certain bacterial taxa regardless of their abundance; confirming the key importance of bacterial activity over taxonomic abundance in controlling caries pathogenesis. The early biomarkers they reported are crucial for understanding the etiology and dynamics of dental caries and devising timely intervention strategies to prevent disease progression.

The Edlund group published the first characterization of the microbe-host immunological marker co-occurrences in dental caries using stimulated and unstimulated saliva (Baker et al., 2021). They used deep metagenomic sequencing to identify novel taxa at the strain-level and surveyed host immunological markers and potential crosstalk between oral bacteria and these markers during caries. They *de novo* assembled 42 novel species, and found that *Prevotella* spp., *Streptococcus mutans*, and Epstein-Barr virus were prevalent in children with caries. Alongside, they identified 10 significantly elevated host immunological markers in the caries group - EGF, IL10, CSF3, IL1RN, IL15, TGF, CSF2, CCL22, IL13, and IL6 - providing an atlas of potential relationships between microbes and host immunological molecules. Further investigations of the relationship between the caries-associated immunological biomarkers and specifically abundant taxa will improve the understanding of the crosstalk between the oral microbiota and the host immune system (Baker et al., 2021).

## Authors Remarks for Future Effort

Omics techniques have certainly provided insights into the dental caries hypotheses and provided new understanding of dental caries etiology and progression over the past 20 years. Even so, reviewing the current state of dental caries research has revealed that there are still significant deficiencies in various aspects that might explain the unsolved mystery of this disease. These deficiencies can be explained only in terms of a complex interplay among the host, microbe, and risk factors. Moreover, the current reports show that untreated dental caries in adult teeth is still ranked #1 among 328 assessed health-related conditions (GBD 2016 Disease and Injury Incidence and Prevalence Collaborators., 2017; U.S. Department of Health and Human Services., 2021). Thus, it is fair to acknowledge that additional modern omics studies might not directly improve the rates of caries prevention or provide a cure, and that improved outcomes might otherwise be achieved by controlling frequent carbohydrate consumption, maintaining good oral hygiene or implementing simple preventive services. For example, promoting prevention programs, such as fluoride varnish, resulted in a historical reduction of early childhood caries by nearly 50% in preschool children as reported in the NIH 2021 executive summary (U.S. Department of Health and Human Services., 2021). Similarly, the widespread application of dental sealants, another preventive aid, has led to notable reductions of teeth loss; only 13% of seniors are edentulous today, compared to 50% in the 1960s (U.S. Department of Health and Human Services., 2021). However, we can claim that the in-depth mechanistic knowledge of caries pathogenesis is moving us closer to the promise of personalized dentistry, in which specific therapies can be individually tailored for treatment and prevention. Herein, we summarize our insights on additional investigations in the following areas and further refinement of implementing these technologies that could demystify the intricacies of this disease:

### A) Standardizing the Definitions of Health and Disease Stages

Given the dynamic and site-specific nature of dental caries, the use of inconsistent and indefinable standards for health and/or disease stages during sampling will result in more questionable conclusions or even contradictory findings (Cugini et al., 2021). No technology, no matter how innovative, can advance our understanding of this disease until we come to a unified and well-defined health and dysbiotic states and stages for both governing sides - the host and the microbiota. Furthermore, these standardized definitions will aid in answering the “chicken or egg” question inherent in all microbial shift disease research by linking the findings to the precise disease state, whether it be caries risk, caries onset, or progressive carious lesions. Correspondingly, new preventive strategies and targeted therapeutics would also be designed and tested on a strong foundation.

### B) Designing Longitudinal Studies at a Large Scale

There is a universal agreement that well-designed longitudinal studies are imperative in reaching meaningful conclusions and that a significant number of studies have relied on small, cross-sectional cohorts. The longitudinal design is particularly valuable in identifying changes in preclinical stages and observing the etiological milieu, which are key to identifying the associated early biomarkers of disease (Gomez and Nelson, 2017; Rosier et al., 2018; Belstrom, 2020; Moussa et al., 2022). Equally important is the large sample size, especially in genetic-linkage studies and detection of genetic risk factors, as a sufficient sample size is necessary to discern the influence of the cultural and genetic factors impacting health and disease (Nibali et al., 2017). The high cost was one of the major interfering factors for such large-scale studies; however, most of omics techniques are now less costly than ever before.

### C) Expanding the Mechanistic Studies on Bacterial Interactions

In dysbiotic conditions, the selective pressure for acidogenic and aciduric microorganisms, prompted by the repeated exposure to free sugars and carbohydrates, is well established in the literature (Takahashi and Nyvad, 2011; Marsh and Zaura, 2017; Bowen et al., 2018). However, further mechanistic studies are still required to understand how microorganisms interact with each other in a polymicrobial synergy, how they develop and become functionally specialized in such a dynamic biofilm form the caries onset to overt lesions, and why the microbial composition may vary on different sites of the tooth surface (Lamont et al., 2018; Bowen et al., 2018). Adaptive bacterial associations can arise from several factors, such as complex signalling, cross-feeding interactions, altered inflamed microenvironment, or other interferences with signalling pathways that govern cell viability, proliferation, and differentiation (Takahashi, 2015; Lamont et al., 2018; Bowen et al., 2018).

Consequently, the conception of the commensal and pathogenic nature of many bacteria has been regarded as too restrictive recently (Whitmore and Lamont, 2011; Nelson and May, 2017). For example, Oral streptococci of the Mitis group (including *Streptococcus gordonii*) was considered a strict commensal. However, it has been found that *Streptococcus gordonii* is an accessory pathogen in mixed infections with *Porphyromonas gingivalis*, which showed increased alveolar bone loss compared to infection with *Porphyromonas gingivalis* alone (Kuboniwa et al., 2017). Likewise, the *Streptococcus mitis* has also been shown to enhance the virulence of *Candida albicans* by promoting fungal tissue invasion and increasing mucosal infection severity (Xu et al., 2014; Bertolini et al., 2015). Thus, polymicrobial infections, involving functionally specialized organisms, are inherently more complex than infections caused by single species, which complicates the study of dental caries pathogenesis. Further integration of omics datasets with the mechanistic studies of phenotype-pathogenicity association is warranted to identify the mechanisms governing oral polymicrobial synergy and dysbiosis and to aid in designing innovative therapeutic approaches (Surana and Kasper, 2017; Lamont et al., 2018).

### D) Considering the Non-Bacterial Oral Microbes

There is no doubt that bacteria dominate the oral microbiome; however, fungi and bacteriophages, or bacterial viruses, are appreciable components of the oral microbiome that impact caries pathogenicity (Willis and Gabaldon, 2020; Yahara et al., 2021). Nevertheless, very little is known about their role during the stages of dental caries disease. Concerns about the collection of fungal genetic material and categorization of fungal species are among the difficulties that have resulted in scarce investigations of this segment of the microbiome (Willis and Gabaldon, 2020). Likewise, very little is known about alteration of oral phage communities although it is known that phages modulate the oral bacteriome, and thus its function (Yahara et al., 2021). Virome studies have increased rapidly to characterize “healthy gut phageome” and to explore the associations between the virome alterations and diseases (Han et al., 2018; Garmaeva et al., 2019). Only a small number of virome studies focused on saliva and subjects with periodontal disease have been done (Ly et al., 2014) and, to our knowledge, no study has investigated the virome in the dental caries setting. Indeed, virome research is difficult since there are no conserved marker regions such as the 16S rRNA gene in bacteria or the nuclear ribosomal RNA cistron in fungi (Willis and Gabaldon, 2020). However, these issues have begun to be resolved by NGS technologies, especially with shotgun long-read metagenomic techniques (Donovan et al., 2018; Yahara et al., 2021). Accordingly, expanding the focus to include other domains of life that are present in the microbiome is necessary, as it generates more extensive knowledge of its taxonomic composition and functionality with deeper understanding of the disease undercurrents.

### E) Effective Integration of Multi-Omics Datasets

Working in silos would not aid in delineating the linkages, correlations, and inter-relationships necessary for a deeper understanding of the behavior of microbiota and host responses during the various stages of dental caries. It is obvious that integrative-omic studies are deficient in dental caries research, particularly between host expression profiles and microbiota behavioral patterns (**Figure 4**), which is the avenue to take to deeply understand the host-microbe interactions and crosstalk (Solbiati and Frias-Lopez, 2018). The scarcity of these studies may explain why the caries disease is still not well-understood. Special attention is needed in the areas of metabolomics and host genetics of caries susceptibility, which are minimally represented in the current research (**Figure 4**), despite being critically needed for preventive personalized dentistry. Also, we believe that the development of polymicrobial models of dental caries, with integrative multi-omics, holds the key for a profound understanding of the disease dynamics, including early risk assessment (Moussa et al., 2022). Biological networks are very promising for downstream analyses of multi-omics datasets and to model interactions between biological entities, such as gene regulation, metabolic and signaling pathways, and protein–protein networks (Aguiar-Pulido et al., 2016). Albeit the continuing efforts in data management and in the broader machine learning communities, considerable efforts are still required to develop optimized and robust pipelines to achieve high analytical accuracy in these massively-large datasets (Mansour et al., 2018).

### F) Implementing Systems Biology Models

Systems biology is defined by the NIH as an approach to understand the larger picture by putting its pieces together (Wanjek, 2011). Despite various systems biology or modeling approaches being applied to investigate microbe–microbe, host–microbe interactions or even extended to investigate an entire microbial community (Thiele et al., 2013), dental caries research has not tackled this direction yet. A key goal of systems modeling is to identify the critical control mechanisms regulating the system’s functions and predict outcomes of testing in experimental settings *via* providing high-throughput simulations (Metzcar et al., 2019).

Dental caries data are typically acquired from various sources including the molecular level (e.g., the different host and microbiota omics datasets), the habitat level (e.g., saliva, AEP, dental plaque, carious lesions), and the clinical level (including the disease stage and inter-patient variability). Coordinating data from these levels allows us to predict a type of dynamic behavior and delineate complex regulatory activities such as positive or negative feedback loops (Tyson and Novak, 2020). This integration can also be staged by developing an initial model framework of the overall system, by considering each level individually, then piecing all levels together to begin rebuilding the overall system (Clarke et al., 2020).

New and improved models continue to evolve rapidly, and they can be either mathematical (algorithms-based) or computational (machine learning-based), mostly using low and high dimensional data, respectively (Clarke et al., 2019). Although the integrative analysis of heterogeneous, large-scale omics data sets is still the grand challenge of systems biology, integrative modeling can adaptively use both approaches, as it leverages the specific hypothesis and data available. In particular, it combines the realistic limits of the number of parameters that can be included effectively in the mathematical models with discovering new features from the computational high dimensional data (Clarke et al., 2020). Additionally, the limitations in data availability, quality and/or uncertainties in mechanistic aspects can be managed by using hybrid modeling approaches (Molinelli et al., 2013; Kraikivski et al., 2015; Tyson et al., 2019; Tyson and Novak, 2020). Furthermore, mathematical modeling can help us to design the next set of experiments to be pursued. Then, based on the new experimental data, modeling can be further refined and improved (Clarke et al., 2020). Collectively, these multiscale integrative systems biology models can drastically improve our holistic understanding of this complex disease and assist in unraveling mechanisms underlying physiological and pathological states.

## Author Contributions

Conceptualization: DM, TM, WS. Data curation: DM, PA. Formal analysis: DM, PA, TM. Investigation: DM, TM. Writing-original draft: DM. Writing-review and editing: DM, PA, TM. Revision and submission approval: all authors. All authors contributed to the article and approved the submitted version.

## Funding

This work was supported by the Canadian Institutes of Health Research (CIHR grant number 341315 and 106657).

## Author Disclaimer

The funding bodies had no role in study design, analysis, and interpretation of data; in the writing of the report; and in the decision to submit the article for publication. The content is solely the responsibility of the authors and does not necessarily represent the official views of the Canadian Institutes of Health Research.

## Conflict of Interest

The authors declare that the research was conducted in the absence of any commercial or financial relationships that could be construed as a potential conflict of interest.

## Publisher’s Note

All claims expressed in this article are solely those of the authors and do not necessarily represent those of their affiliated organizations, or those of the publisher, the editors and the reviewers. Any product that may be evaluated in this article, or claim that may be made by its manufacturer, is not guaranteed or endorsed by the publisher.
